# Associations of latitude and photoperiod with sleep duration in a yearlong study of US physicians

**DOI:** 10.1016/j.sleep.2025.106840

**Published:** 2025-10-03

**Authors:** Katherine ET. Ross, Karina Pereira-Lima, Kerby Shedden, Margit Burmeister, Srijan Sen

**Affiliations:** a Department of Psychology, University of Michigan, Ann Arbor, MI, 48104, USA; b Michigan Neuroscience Institute, University of Michigan, Ann Arbor, MI, 48109, USA; c Department of Anesthesiology, University of Michigan, Ann Arbor, USA; d Department of Statistics, University of Michigan, Ann Arbor, MI, 48109, USA; e Departments of Computational Medicine and Biochemistry, Psychiatry, and Human Genetics, University of Michigan, Ann Arbor, USA; f Eisenberg Family Depression Center, University of Michigan, Ann Arbor, MI, 48109, USA; g Michigan Medicine, Department of Psychiatry, Ann Arbor, MI, 48109, USA

**Keywords:** Sleep, Sleep duration, Geography, Photoperiod, Seasonal variation

## Abstract

Geographic factors may influence sleep, but prior studies have been inconsistent, likely because of reliance on seasonally invariant and self-reported data. Using year-long objective sleep measurements from 4683 U S. medical interns, this study aimed to determine how latitude affects total sleep time (TST) and assess whether photoperiod acts as a mediator in this association. Linear mixed models (LMMs) showed that each 1° increase in latitude was associated with approximately 0.23 min longer TST on average across the year, and daily photoperiod was negatively associated with TST. Simulation-based mediation analyses revealed seasonal effects: in summer, higher latitude led to reduced TST via longer photoperiod, whereas in winter, higher latitude led to increased TST via shorter photoperiod. Direct effects of latitude remained significant during winter, suggesting additional influences beyond photoperiod. These findings indicate that latitude influences TST through a seasonally specific photoperiod-mediated pathway, as well as through other non-photoperiod environmental factors.

## 1. Introduction

Sufficient sleep duration is crucial for cognitive function, emotional regulation, and overall physiological health [1,2]. Despite its importance, a large proportion of Americans get insufficient sleep, with 36.8 % of US adults sleeping less than 7 h per night in the 2022 Behavioral Risk Factor Surveillance System (BRFSS [3]). Understanding the environmental factors that influence sleep patterns is crucial for designing effective interventions.

Natural light suppresses melatonin production, increasing propensity for wakefulness [4], and regulates circadian rhythms, helping to synchronize our internal sleep-wake patterns to the external day-night cycle [5]. Social schedules, such as work hours, school times, can operate discordantly to this natural light-dark cycle, creating a misalignment between biological and social time that can contribute to sleep loss [6]. Photoperiod, or the duration of natural light each day, varies across the year, with longer days in the summer and shorter days in the winter in the Northern Hemisphere. There is generally consistent evidence that sleep duration tends to increase in winter compared to summer [7–9]. Recent research has also shown seasonal shifts in sleep timing (e.g., midsleep) and relatively stable TST in university students; however, this study used different cohorts in each season, and was only able to sample the winter cohort once [10].

Given the influence of light on sleep, latitude, which affects seasonal and daily photoperiod may have significant effects on sleep patterns. Higher latitudes have greater seasonal variation in photoperiod, with longer days in the summer and shorter days in the winter compared to constant photoperiod at the equator. However, findings on the impact of latitude itself on sleep duration have been mixed. Existing studies rely on self-reported data across limited time spans, limiting their ability to capture the seasonally varying and nuanced effects of latitude. For example, one study performed in Chile during the spring found that individuals living at higher latitudes (further from the equator) self-reported longer sleep duration compared to those closer to the equator [11]. However, this study relied on a single-point measure of sleep duration, total annual sunlight, and dichotomized outcomes (short versus long sleepers) at categorical intervals of latitude, precluding the ability to quantify how much total sleep time (TST) changes as a function of latitude or photoperiod length.

In contrast, another study among Turkish students [12], which measured sleep between December and March, found no significant association between latitude and overall sleep duration. However, this study may have been underpowered to detect effects of latitude given the narrow geographic span (~6° compared to ~20° in the [11]). This research also relied on self-reported sleep schedules and was limited to a short timeframe, again precluding the capture of seasonally varying effects. These conflicting findings underscore the need for studies utilizing sleep assessments, geographically varying samples, and longitudinal, multi-seasonal design to understand how latitude and photoperiod are associated with TST across different times of the year.

Here, using objective sleep data collected from wearable devices in a large, multisite, year-long longitudinal cohort study, we assessed the associations between latitude and daily TST and whether photoperiod mediates this association. By addressing limitations in prior research, our study allows us to examine how latitude influences sleep duration differently across seasons, which has not been systematically explored in prior studies.

## 2. Methods

### 2.1. Participants

The Intern Health Study follows training physicians through the first year of medical residency (i.e., internship) (for details, see Ref. [13]). A total of 20,668 interns starting residency between 2018 and 2022 were invited to participate in the Intern Health Study, 8927 (43.19 %) of whom enrolled. Prior to the start of internship (July 1 of cohort year), subjects completed a baseline survey assessing demographic and program data through a mobile app. Subjects also wore Fitbit devices to measure sleep continuously from time of enrollment through the internship year. This study was approved by the University of Michigan Institutional Review Board and all subjects provided informed consent and received a Fitbit and between $25 to $130 in compensation.

### 2.2. Measures

We determined geographic location through each intern’s residency institution address (https://simplemaps.com/us). Fitbit-based metrics for each sleep episode included time asleep in minutes and bed and wake time with 30s granularity. Daily TST was calculated by summing the duration of all sleep episodes with a heart rate upon waking for a 24-h period. This criterion was used to exclude episodes where signal loss (e.g., due to battery depletion or device removal) might falsely mark the end of sleep, resulting in inaccurate TST estimates. The episode with the greatest amount of measurement time between start and finish was considered the main sleep episode and used for daily-level bed and wake times. To improve the reliability of model estimates and reduce the influence of extreme values, we identified and removed outliers using a z-score threshold of ±3 standard deviations from the mean. To reduce artifacts potentially related to shift work, we only included sleep data where the main episode began between 6:00 p.m. and 6:00 a.m. and the window between bed and wake time lasted at least 4 h [14]. Interns with missing residency institution locations and less than 7 days of Fitbit sleep data were excluded from analyses. Because enrollment and data completeness may vary systematically by demographic or specialty factors, which could bias estimates of geographic or seasonal effects on sleep, we applied scaled weights that accounted for both demographic representativeness and wearable data completeness. Post-stratification weights were generated to align our sample with national distributions of U.S. resident physicians by gender, race/ethnicity, specialty, and cohort year. Attrition weights were derived using propensity scores for contributing sufficient valid wearable data, based on demographic and specialty characteristics. Final weights were the product of these components and were scaled to the analytic sample size (see eAppendix 2 in Supplement 1 in Ref. [15]).

### 2.3. Linear mixed models

We employed 3-level nested linear mixed-effects models (LMMs) to examine the effects of latitude on TST. Random intercepts were specified at both the internship program and individual levels to account for the hierarchical nature of the data (daily sleep nested within individuals, individuals nested within internship programs).

We first fit a model examining the unadjusted effect of latitude on TST. Prior to fitting a model with covariates, we assessed the potential for multicollinearity by computing bivariate correlations among all candidate predictors with latitude and photoperiod. Following Dormann et al. (2013), we excluded variables that exceeded a threshold of |r| > 0.70. We then fit an adjusted model including latitude and remaining covariates. Covariates considered included East-West geographic position within time zone (PTZ), temperature, age, sex, race/ethnicity, surgical status, internship day, sleep midpoint, and type of day (weekday/weekend). PTZ was included because variability in photoperiod timing within a time zone, driven by East-West position, may affect sleep duration independently of latitude [8,16–18]. PTZ was computed as each participant’s longitudinal distance from the central meridian of their respective U.S. time zone (Eastern = −75°, Central = −90°, Mountain = −105°, Pacific = −120°). These values correspond to the standard central meridians used to define U.S. time zones and approximate solar noon, thereby allowing PTZ to serve as a proxy for misalignment between solar and social time.

Temperature, age, sex, and race/ethnicity were included because of their established associations with sleep [19,20]. Type of day was a dichotomous variable where Monday-Friday = 0, Saturday and Sunday = 1 [21]. Days on internship were included to control for potential changes in sleep patterns across the year due to the unique stressors and demands of medical training. Sleep midpoint, often used as a surrogate for phase, a crucial modifier of TST, was calculated as the midpoint between bed and wake time of the main sleep episode and reported as minutes after midnight [18,22]. Given the possibility for sleep midpoint to be on the causal pathway from latitude and/or photoperiod to sleep duration (e.g. Ref. [5,18]), we ran a sensitivity analysis excluding sleep midpoint to ensure that adjusting for it did not bias our final interpretation.

To further assess the robustness of our findings, we conducted sensitivity analyses for all aforementioned LMMs using a subsample of participants with broader seasonal data coverage. Specifically, we limited this analysis to individuals with at least seven days of valid sleep data in each of four 3-month seasonal windows: near the summer solstice (May–July), winter solstice (November–January), spring equinox (February–April), and fall equinox (August–October).

### 2.4. Mediation analysis

To assess whether photoperiod mediates the relationship between latitude and TST, we used a model-based simulation approach using a refit LMM including both latitude and photoperiod as predictors. Because the relationship between latitude and photoperiod is deterministic but strongly moderated by day of year, traditional mediation models were not appropriate. Instead, we used a bootstrapped simulation-based approach motivated by this nonlinearity. Using fixed-effect estimates from the adjusted LMM, we simulated TST under counterfactual scenarios in which latitude and photoperiod were manipulated independently or jointly using the following equations:

Photoperiod-mediated (how changes in latitude affect sleep through changes in photoperiod; the difference in sleep between the scenarios where only photoperiod is varied):

$$\mathrm{sleep}(\mathrm{photoperiod}({\mathrm{lat}}_{1},\;\mathrm{doy}),\;{\mathrm{lat}}_{0})-\mathrm{sleep}(\mathrm{photoperiod}({\mathrm{lat}}_{0},\;\mathrm{doy}),\;{\mathrm{lat}}_{0})$$

Non-photoperiod mediated (how latitude influences sleep independently of photoperiod; the difference in sleep between the scenarios where latitude varies and photoperiod is held constant):

$$\mathrm{sleep}(\mathrm{photoperiod}({\mathrm{lat}}_{0},\;\mathrm{doy}),\;{\mathrm{lat}}_{1})-\mathrm{sleep}(\mathrm{photoperiod}({\mathrm{lat}}_{0},\;\mathrm{doy}),\;{\mathrm{lat}}_{0})$$

Total effect (overall change in sleep when both photoperiod and latitude change):

$$\mathrm{sleep}(\mathrm{photoperiod}({\mathrm{lat}}_{1},\;\mathrm{doy}),\;{\mathrm{lat}}_{1})-\mathrm{sleep}(\mathrm{photoperiod}({\mathrm{lat}}_{0},\;\mathrm{doy}),\;{\mathrm{lat}}_{0})$$

We chose two latitudes to contrast: lat_0_ = 35° and lat_1_ = 45°, which fall near the central range of the U.S. sample. Simulations were run for June 20, 2020 (2020 summer solstice) and December 21, 2020 (2020 winter solstice) to represent seasonal extremes. We also generated 95 % confidence intervals for the photoperiod-mediated, non-photoperiod mediated, and total effects of latitude on TST based on 100 parametric bootstrap samples. Random effects from the LMM controlled for individual and institutional variability, and counterfactual photoperiod under different latitudes was calculated from known geophysical formulas using the suncalc package in R, while sleep duration under these conditions was imputed using the fixed effects from the LMM. We examined the association between latitude and TST using a generalized additive model (GAM) to assess whether the results could reasonably generalize across the observed latitude range.

This approach allows us to partition the association between latitude and TST into a component shared with photoperiod and a residual component that may reflect other latitude-related factors (e.g., cultural, behavioral, or environmental features not captured by photoperiod). Analyses were conducted in R with a 2-sided p < .05 and 95 % CI excluding 0 deemed as statistically significant.

## 3. Results

### 3.1. Sample demographics

**Fig. 1.**
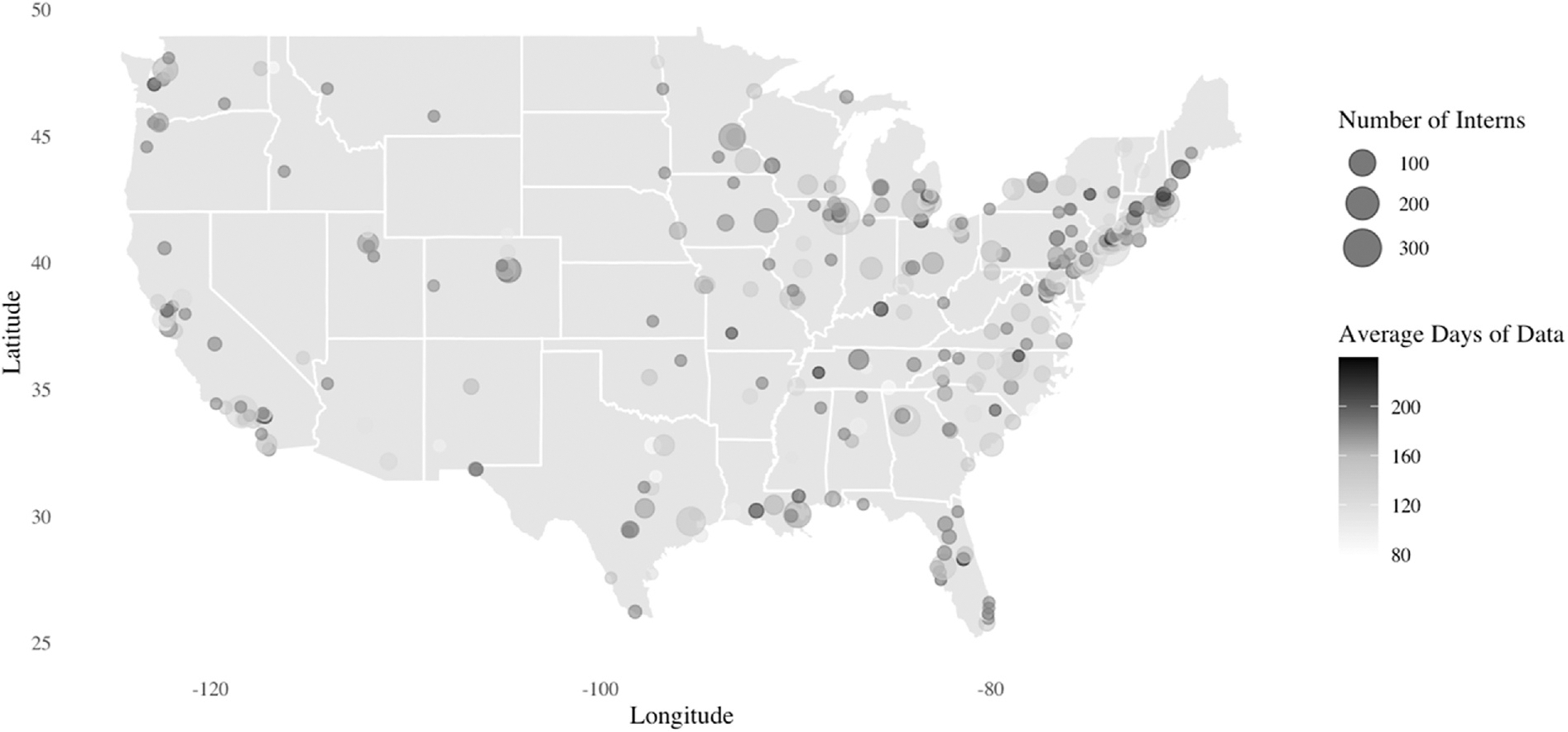
Geographic Spread of Data Note. Each point represents a U.S. residency location, colored by the average days of data provided across all interns at that location throughout the year. Darker colors indicate more days of sleep data. Larger size indicates greater number of interns at each location. Locations are plotted by longitude and latitude, showing spatial variation in average TST across the continental U.S. This figure demonstrates geographic spread and visually supports a modest geographic gradient, with slightly longer TST observed at higher latitudes.

Initial heart rate validation resulted in a 811,518 daily observations across 5332 interns. Removing outliers with TST values greater than 3 standard deviations from the sample mean removed 13,259 (1.6 %) observations, limiting the range of daily TST to between 95.44 and 714.47 min (1.59 and 11.91 h) across 5309 interns. Further restricting to overnight episodes (those beginning between 6pm and 6am and lasting ≥4 h) removed an additional 90,493 (11.15 %) observations and reduced the analytic sample to 5240 interns. A final sample of 4683 interns with ≥7 nights of sleep data and complete demographic information were included in the current analysis. Subjects were a slight majority female (2561, 54.7 %), White (2677, 57.2 %) and had an average of 416.43 (83.64) minutes (6.94 h) of daily TST (full demographics in Table 1). Interns had an average of 148.60 (109.13) days of sleep data (range = 7–424), and each day of the year had an average of 1905.53 (627.30) interns providing sleep data (excluding 2/29, which only occurred for the 2019 cohort; Fig. 1 of Supplement). Interns spanned a broad geographic region across the US (latitude range = [25.78, 48.08], longitude range = [−123.28, −69.73]; see Fig. 1).

### 3.2. Linear mixed models with latitude predicting TST

**Fig. 2.**
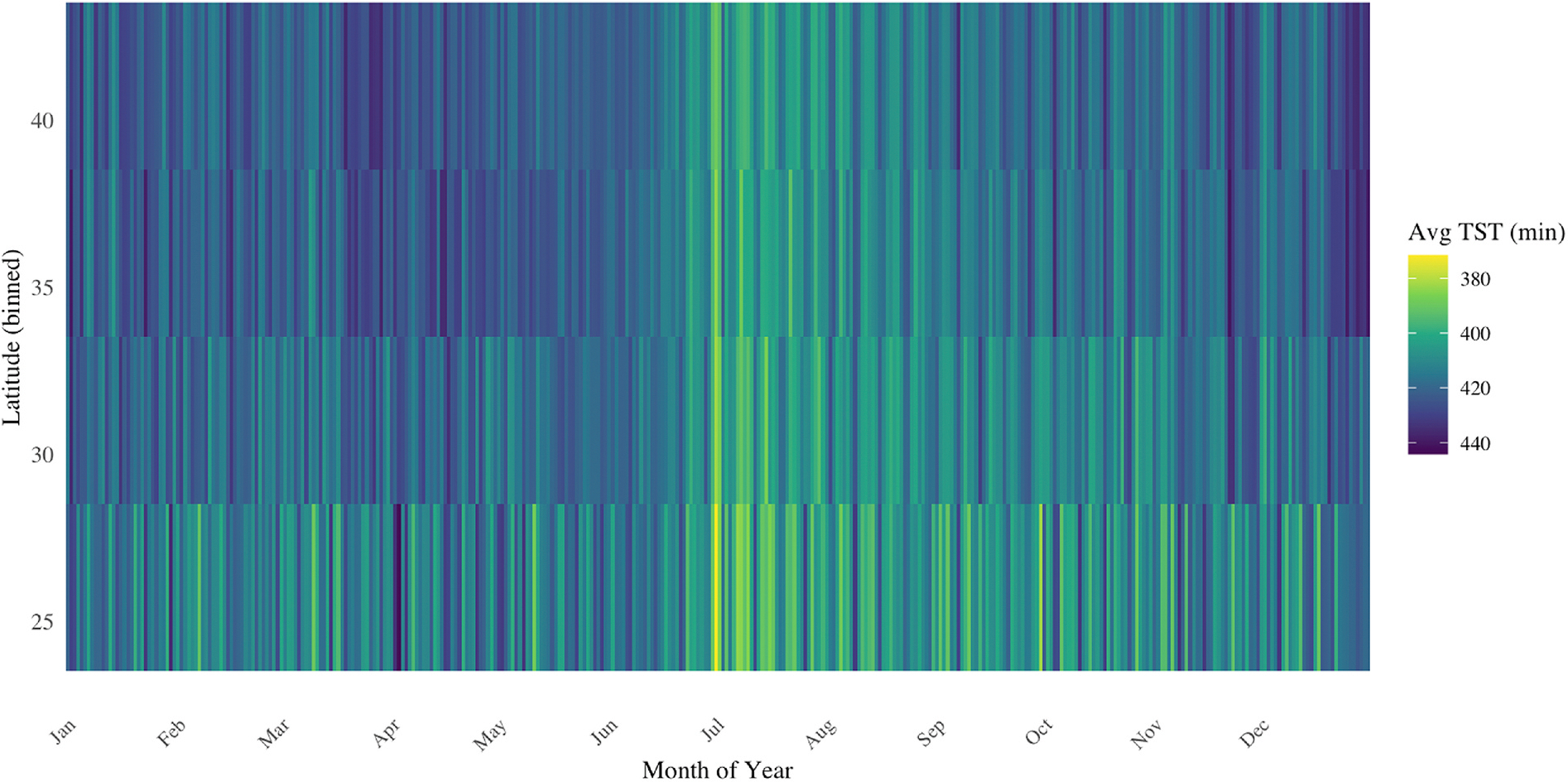
Variation in TST by Latitude Across Calendar Year Note. Heatmap displaying average daily total sleep time (TST, minutes) across the calendar year (x-axis) by 5° latitude bins (y-axis). Darker colors indicate longer sleep duration. (For interpretation of the references to color in this figure legend, the reader is referred to the Web version of this article.)

Photoperiod and temperature were highly correlated (r = 0.71); therefore, temperature was excluded from the final models. Results from the LMMs are displayed in Table 2 and Fig. 2. In all LMMs, beta coefficients represent estimated changes in total sleep time (TST) in minutes per 1-unit increase in continuous predictor variables, or change compared to reference group in categorical predictor variables. In the unadjusted LMM, latitude was associated with TST (b = 0.43, p = .001), indicating that 1° of latitude increase is associated with an average of 0.43 additional minutes of sleep per night across the year (Fig. 2).

In the adjusted LMM that included demographic and occupational covariates, the effect of latitude on TST was attenuated and not statistically significant (b = 0.19, p = .09). In contrast, several demographic and professional covariates demonstrated larger and statistically significant associations with TST. For example, female interns slept an average of 19.74 min longer than males, Black and Asian interns slept approximately 24.98 and 21.30 min less than White interns, respectively, and interns in surgical specialties slept an average of 8.60 min less than those in nonsurgical specialties (Table 2). Sensitivity analyses among the subset of participants with broader seasonal coverage across the year (N = 2211), revealed no major changes in result interpretation, although the effect of latitude was statistically significant in the covariate-adjusted model (Supplemental Table 1). Although the effect of latitude was attenuated across the year, we proceeded with models testing photoperiod as an explanatory pathway due to its conceptual role as a primary environmental mechanism linking latitude to annual variation in sleep.

Additional sensitivity analyses excluding sleep midpoint also revealed no major changes in result interpretation. Notably, when midpoint was excluded the effect of latitude remained statically significant in covariate-adjusted models in both the full sample and the subsample with broader seasonal coverage (Supplemental Tables 2 and 3).

### 3.3. Mediation analysis

**Fig. 3.**
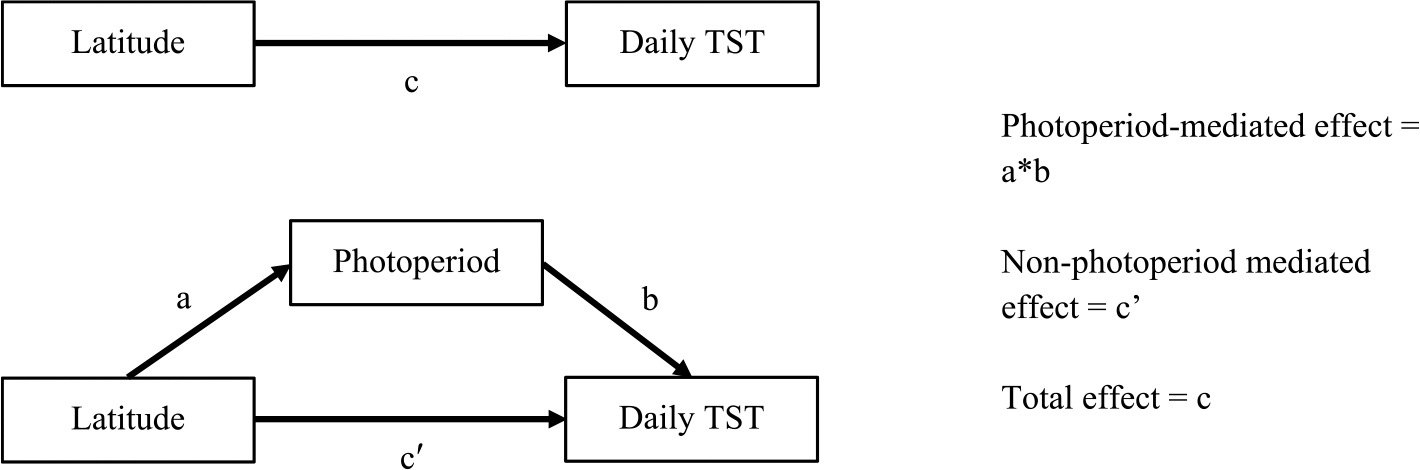
Conceptual Mediation Model Note. Conceptual mediation model illustrating the relationship between latitude and total sleep time (TST) through photoperiod. The top panel depicts the total effect of latitude on TST. Bottom panel illustrates the photoperiod-mediated (indirect) effect through daylight duration (path a * b), and the non-photoperiod mediated (direct) effect of latitude on TST (path c′).

The refit model including latitude, photoperiod, and other covariates revealed similar estimates for the effect of latitude (b = 0.25, p = .03) on TST. Photoperiod showed a small, negative effect on TST (b = −0.04, p < .001). Using the resulting coefficients from this LMM including latitude, photoperiod, and all covariates, we assessed whether photoperiod mediates the association between latitude and TST (see Table 2; Fig. 3; Table 3).

Visual inspection of the GAM revealed an approximately linear association between latitude and TST across our range of observed latitudes (Supplemental Fig. 2), suggesting generalizability of the mediation results across our sample. During the summer, the association between latitude and TST was significantly mediated by photoperiod (b = −2.35, SE = 0.05, 95 % CI: [−2.44, −2.22]), such that a 10-degree increase in latitude (approximately equivalent to moving from Atlanta, GA to Detroit, MI) resulted in −2.35 min shorter TST due to longer photoperiod hours. During the winter, the direction of the photoperiod-mediated effect reversed (b = 2.20, SE = 0.05, 95 % CI: [2.12, 2.31]), where a 10-degree increase in latitude was associated with 3.91 min longer TST due to shorter photoperiod.

The non-photoperiod-mediated (i.e., direct) effect of latitude was positive but non-significant in summer (b = 2.43, SE = 1.32, 95 % CI: [−0.12, 5.15]), and positive and significant in winter (b = 2.51, SE = 1.03, 95 % CI: [0.43, 4.23]), suggesting a modest residual influence of latitude on TST independent of photoperiod, particularly during winter months.

The total effect of latitude on TST was positive and significant during winter (b = 4.71, SE = 1.02, 95 % CI: [2.60, 6.44]), indicating a combined photoperiod-mediated and non-photoperiod mediated effect of latitude on TST of nearly 5 min per 10 degrees of latitude. The total effect of latitude on TST during summer was near zero and non-significant (b = 0.08, SE = 1.32, 95 % CI: [−2.41, 2.81]).

## 4. Discussion

By using objective, longitudinal sleep data collected across a full year, this study clarifies prior inconsistencies regarding how latitude affects sleep duration. We found that higher latitude is associated with longer TST, even after adjusting for photoperiod and other covariates. Specifically, each 1° increase in latitude is linked to approximately 0.25 additional minutes of TST, suggesting a modest but significant effect of geographic location on sleep behavior.

Our mediation analysis also reveals that this relationship is partially mediated by photoperiod in a seasonally dependent manner. In the summer, longer photoperiods at higher latitudes are associated with reduced TST, while in winter, shorter photoperiods are associated with increased TST. This indicates that reduced light in winter facilitates longer sleep, consistent with theories of photoperiodic regulation of circadian and sleep processes [4,5]. However, the persistence of a direct effect of latitude even after accounting for photoperiod indicates that other geographically patterned factors may contribute to variation in sleep. For example, greater annual variability in photoperiod, beyond the day-to-day duration of light, may shape behavioral rhythms. Additionally, environmental factors that vary seasonally and systematically with latitude such as temperature may influence sleep independent of photoperiod [11]. Although temperature was excluded from our final models due to strong correlation with photoperiod, future work should explore analytic approaches that can better disentangle these overlapping seasonal signals.

The effects of latitude on TST showed consistent seasonal patterns but were modest in magnitude—about 2–5 min of additional sleep per 10° increase in latitude. Several demographic and professional covariates had stronger associations with TST than latitude, including sex, race/ethnicity, and specialty (surgical/nonsurgical). This suggests that while natural environmental factors like photoperiod do influence sleep, their impact is relatively constrained in modern contexts. Human-driven factors such as artificial light exposure, work schedules, and cultural routines may play a more dominant role in shaping sleep behavior. Future research should examine how these social and behavioral influences interact with natural environmental cues to optimize sleep patterns in contemporary environments.

This study has several strengths, including the use of objective sleep data from wearable devices and the availability of this data across the full year, a large geographically diverse sample, and an analytic strategy that isolates the effects of latitude through both photoperiod-mediated and independent pathways. Our simulation-based mediation strategy allowed us to account for the nonlinear, deterministic relationship between latitude and photoperiod while estimating season-specific effects.

However, the study also has limitations. First, the sample consists solely of medical interns, who experience unusually demanding schedules compared to a general population. While we limited sleep episodes to those initiated between 6 p.m. and 6 a.m. to reduce contamination by night shifts, we could not account for potential lingering effects of shift work or sleep debt on TST because data on shift schedules was not captured. These exclusion criteria were applied to increase overall generalizability outside the intern physician population, but may reduce generalizability to interns’ actual sleep patterns, especially those on night rotations. Second, while participants were distributed across the U.S. and the relationship between latitude and daily TST appeared approximately linear, the generalizability of these findings could be limited for populations living at more extreme latitudes (e.g., near the polar regions or equator). Interns contributed approximately six months of sleep data on average, and while sensitivity analyses in a subset with broader seasonal coverage did not reveal notable differences in model estimates, it is possible that limited year-round data may have attenuated seasonal effects or introduced confounding between person-level characteristics and time of year. Third, the study does not account for individual factors such as the use of artificial light or chronotype (morningness vs. eveningness), which may influence the relationship between latitude, photoperiod, and sleep [23]. In addition, we did not directly analyze the effects of daylight saving time (DST), which may interact with chronotype to differentially affect sleep. Prior work using IHS data has shown that DST transitions transiently reduce sleep, with longer-lasting disruption for evening chronotypes compared to morning types (≥1 week vs. 3 days [14]); however it is unlikely that disruptions on this scale impact annual trends. We also did not directly measure light exposure, which is a critical future direction. Recent advances in wearable sensors that capture ambient light may help clarify how much light individuals actually experience, and how light exposure impacts sleep.

Our findings highlight the potential role of geographic and seasonal factors in shaping sleep patterns. On the one hand, the effects we observed were modest in magnitude (~2–5 min of TST per 10° latitude or seasonal contrast), especially relative to demographic and occupational influences. On the other hand, their consistency across a large, geographically diverse sample suggest that geographic context may still contribute meaningfully to population-level variation in sleep. While current sleep recommendations often assume a uniform need across latitudes and seasons, our results raise the possibility that sleep opportunity or need may vary based on environmental context. Future research should investigate whether health outcomes are optimized by stable sleep patterns across the year or by allowing sleep to flexibly align with seasonal environmental changes. Clarifying this relationship could inform how best to support sleep health across diverse geographic settings.

## Supplementary Material

1

2

3

4

5

## Figures and Tables

**Table 1:** Demographic table.

	n (%)				

*Sex*					
M	2122 (45.31)				
F	2561 (54.69)				
Age, mean (sd) [range]	27.66 (2.60) [22, 49]				
*Race/Ethnicity*					
White	2677 (57.16)				
Black/African American	248 (5.30)				
Latino/Hispanic	185 (3.95)				
Asian (e.g. Indian, Chinese)	1055 (22.53)				
Native American	5 (0.11)				
Arab/Middle Eastern	66 (1.41)				
Multi-racial	432 (9.22)				
Other	15 (0.32)				
*Specialty*					
Surgical	861 (18.39)				
Non-Surgical	3822 (81.61)				
*Days of data*	Spring	Summer	Fall	Winter	Total
Days of Data, mean (sd) [range]	40.81 (33.82) [1, 165]	52.61 (27.26) [1, 102]	46.20 (26.58) [1, 91]	43.57 (26.73) [1, 91]	164.43 (121.77) [7, 424]
*Sleep*	Weekday		Weekend	Total	
Total Sleep Time (TST, min), mean (sd) [range]	408.32 (78.93) [100–714]		437.41 (91.47) [97–714]	416.43 (83.64) [97–714]	
Sleep Midpoint, mean (sd) [range]	165.26 (89.76) [−240 – 743]		217.44 (105.21) [−231 – 731]	179.81 (97.18) [−240 – 743]	
*Geographic Position*					
Latitude, mean (sd) [range]	38.69 (4.56) [25.78, 48.08]				
Longitude, mean (sd) [range]	−87.52 (14.33) [−123.28, −69.73]				

Note. Demographic breakdown (sex, race/ethnicity, surgical vs. non-surgical specialty) and seasonal coverage (number of days with valid sleep data in each season) are reported for the final analytic sample (N = 4683). Weekday and weekend TST (minutes) and midsleep (minutes after midnight) are presented separately, along with the overall averages.

**Table 2:** Unadjusted and adjusted LMMs.

Variables	Unadjusted		Adjusted for covariates		Adjusted for photoperiod and covariates	
	b (95 % CI)	p	b (95 % CI)	p	b (95 % CI)	p

Latitude	**0.43 (0.18, 0.68)**	**0.001**	0.19 (−0.03, 0.41)	0.09	**0.25 (0.03, 0.48)**	**0.03**
Photoperiod	–	–	–	–	**−0.04 (−0.04, −0.03)**	**<0.001**
PTZ	–	–	0.03 (−0.22, 0.28)	0.83	0.03 (−0.22, 0.28)	0.83
Age	–	–	**−0.77 (−1.13, −0.42)**	**<0.001**	**−0.78 (−1.14, −0.43)**	**<0.001**
Sex	–	–	**19.74 (17.88, 21.60)**	**<0.001**	**19.70 (17.84, 21.56)**	**<0.001**
*Ethnicity*						
Arab/Middle Eastern	–	–	**−19.23 (−17.07, 14.46)**	**<0.001**	**−19.29 (−27.14, 11.44)**	**<0.001**
Asian	–	–	**−21.30 (−23.61, −18.98)**	**<0.001**	**−21.29 (−23.60, −18.97)**	**<0.001**
Black/African American	–	–	**−24.98 (−29.20, −20.76)**	**<0.001**	**−24.98 (−29.19, −20.76)**	**<0.001**
Latinx/Hispanic	–	–	**−10.62 (−15.44, −5.79)**	**<0.001**	**−10.52 (−15.33, −5.70)**	**<0.001**
Multi-racial	–	–	**−5.53 (−8.71, −2.22)**	**0.001**	**−5.48 (−8.73, −2.24)**	**0.001**
Native American	–	–	−6.65 (−37.79, 21.64)	0.59	−6.27 (−35.93, 23.39)	0.68
Other	–	–	−0.91 (−16.72, 14.89)	0.91	−1.27 (−17.04, 14.49)	0.87
Surgical specialty	–	–	**−8.60 (−11.09, −6.11)**	**<0.001**	**−8.30 (−10.78, −5.81)**	**<0.001**
Days on internship	–	–	**0.03 (0.02, 0.03)**	**<0.001**	**0.02 (0.01, 0.02)**	**<0.001**
Sleep Midpoint (minutes after midnight)	–	–	**0.23 (0.23, 0.23)**	**<0.001**	**0.23 (0.23, 0.24)**	**<0.001**
Weekend	–	–	**17.01 (16.61, 17.42)**	**<0.001**	**16.89 (16.49, 17.30)**	**<0.001**

Note. a. 95 % confidence intervals were computed using the Wald method. b. PTZ = East-west position within time zone, calculated as the difference between institutional longitude and the central meridian of the time zone (e.g., −75° for Eastern, −90° for Central). c. Surgical specialty = binary assignment of surgical specialties were assigned based on the American College of Surgeons classification and included Neurological Surgery, Obstetrics and Gynecology, Ophthalmology, Orthopedic Surgery, Otolaryngology, Plastic Surgery, Surgery-General, Thoracic Surgery, Urology, and Vascular Surgery. Nonsurgical specialties included Anesthesiology, Child Neurology, Emergency Medicine, Family medicine, Internal Medicine, Internal Medicine/Emergency Medicine, Internal Medicine/Pediatrics, Internal Medicine/Psychiatry, Interventional Radiology, Neurology, Pathology-Anatomical and Clinical, Pediatrics, Pediatrics/Medical Genetics, Pediatrics/Psychiatry/Child and Adolescent Psychiatry, Physical Medicine and Rehabilitation, Psychiatry, Psychiatry/Family Medicine, Radiology-Diagnostic, Transitional Year. d. Sleep midpoint is the midpoint of sleep episode, expressed as minutes after midnight on the day the sleep episode ends.

**Table 3:** Mediation analysis results.

	Summer (holding date constant to 6/20)	Winter (holding date constant to 12/21)

Photoperiod-mediated effect	**−2.35 (0.05) [−2.44, −2.22]**	**2.20 (0.05) [2.12, 2.31]**
Non-photoperiod mediated effect	2.43 (1.32) [−0.12, 5.15]	**2.51 (1.03) [0.43, 4.23]**
Total effect	0.08 (1.32) [−2.41, 2.81]	**4.71 (1.02) [2.60, 6.44]**

Note. Table 3 displays the estimated photoperiod-mediated, non-photoperiod mediated, and total effects (b, SE, 95 % CI) of a 10-degree increase in moving from 35° to 45° latitude on TST, stratified by season (Summer: June 20, 2020; Winter: December 21, 2020) and holding date constant. Effects are reported in minutes.

## Appendix A. Supplementary data

Supplementary data to this article can be found online at https://doi.org/10.1016/j.sleep.2025.106840.
