# Supplementary material for: Associations of latitude and photoperiod with sleep duration in a yearlong study of US physicians
Source: Sleep Med. Author manuscript; Available in PMC 2026 Jul 1. (PMC13322165; doi:10.1016/j.sleep.2025.106840)
Supplement: 1 [file NIHMS2184662-supplement-1.docx]

Supplemental Figure 1. User Coverage by Calendar Day


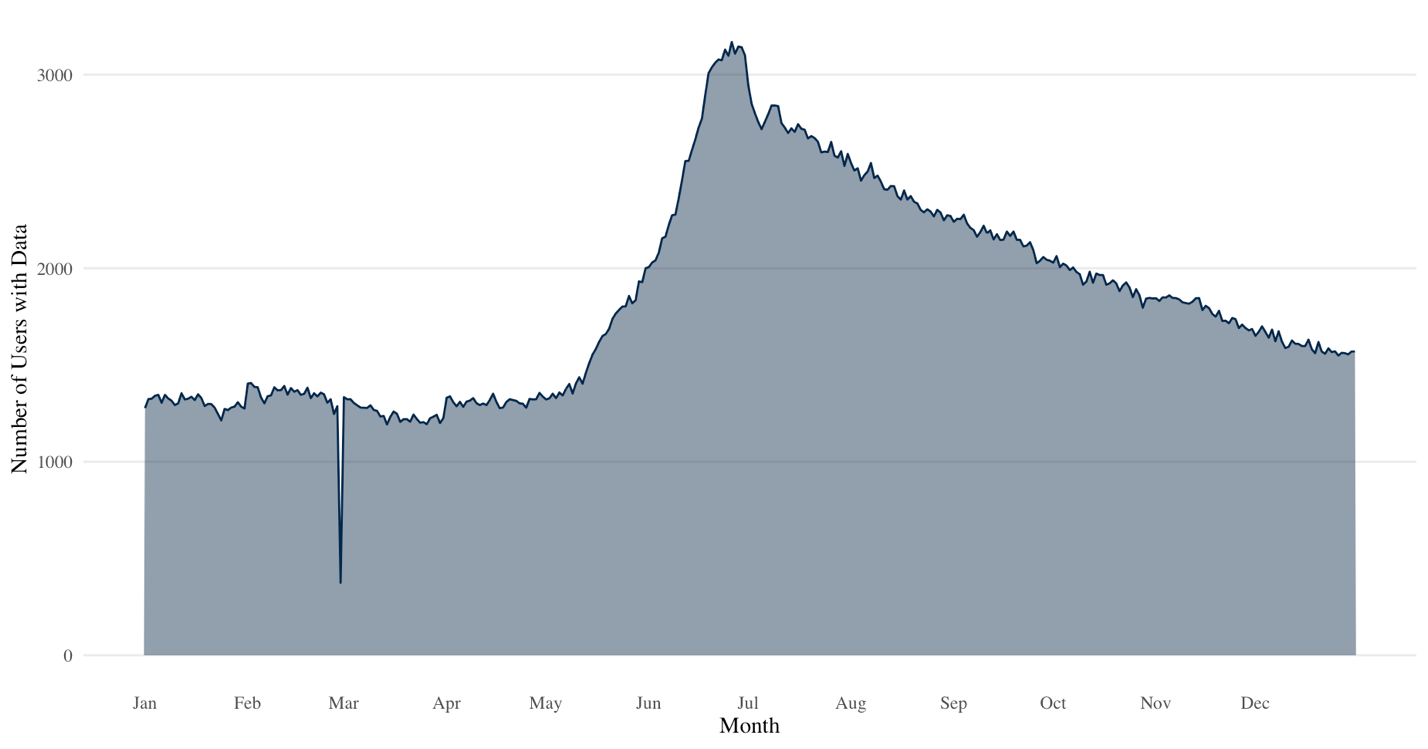


Note. Density plot displaying number of IHS participants with data on each day of the year. The sharp dip in end of February represents 2/29, which only occurs for the 2019 IHS cohort. July 1 marks the start of internship.
