# Supplementary material for: Associations of latitude and photoperiod with sleep duration in a yearlong study of US physicians
Source: Sleep Med. Author manuscript; Available in PMC 2026 Jul 1. (PMC13322165; doi:10.1016/j.sleep.2025.106840)
Supplement: 2 [file NIHMS2184662-supplement-2.docx]

Supplemental Figure 2. Testing Linearity in the Latitude–TST Relationship


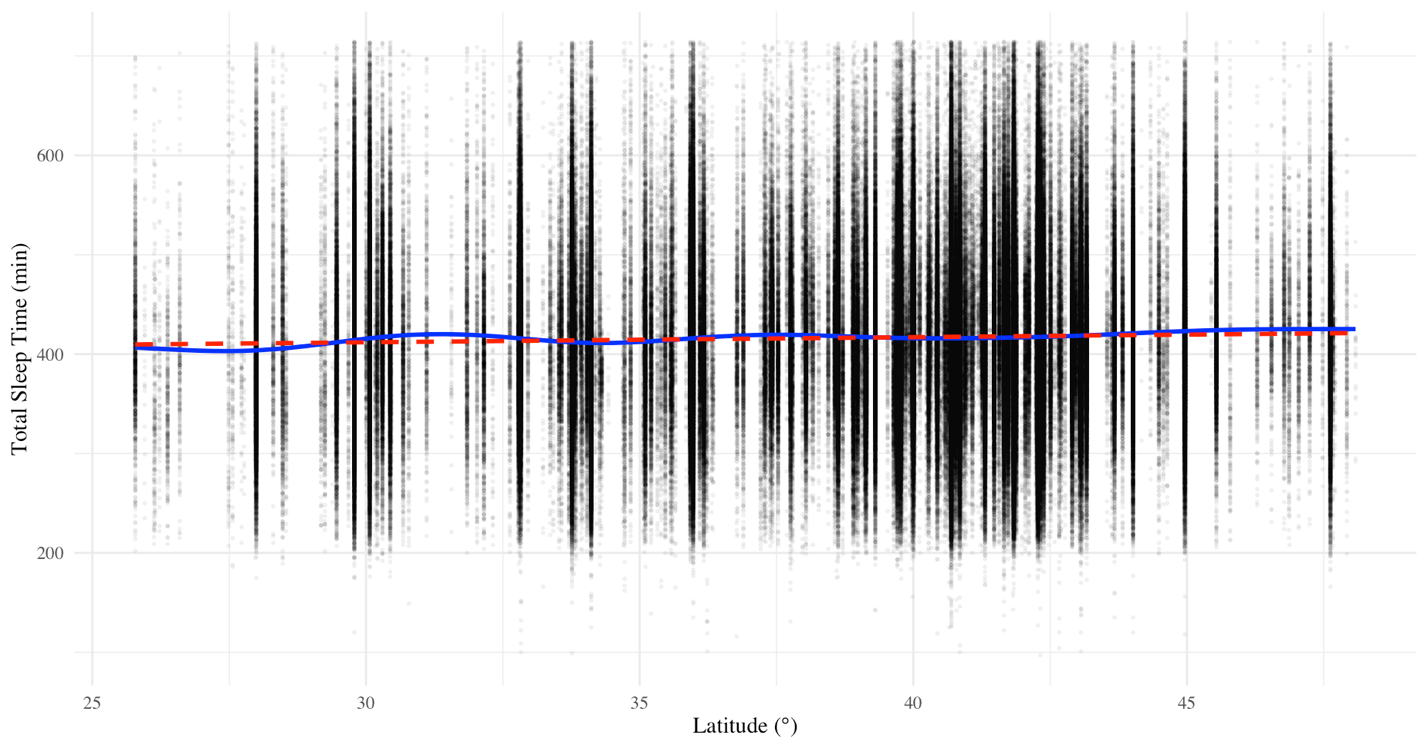


Note. Relationship between latitude and total sleep time (TST). Each point represents one night of TST. The red dashed line represents a linear relationship; the blue line shows a smoothed generalized additive model (GAM) curve with 95% confidence bands.
