# Supplementary material for: Associations of latitude and photoperiod with sleep duration in a yearlong study of US physicians
Source: Sleep Med. Author manuscript; Available in PMC 2026 Jul 1. (PMC13322165; doi:10.1016/j.sleep.2025.106840)
Supplement: 3 [file NIHMS2184662-supplement-3.docx]

Table 1. Unadjusted and Adjusted LMMs Among Interns with Broad Annual Coverage

|  | Unadjusted | | Adjusted for covariates | | Adjusted for photoperiod and covariates | |
| --- | --- | --- | --- | --- | --- | --- |
| Variables | b (95% CI) | p | b (95% CI) | p | b (95% CI) | p |
| Latitude | **0.61 (0.30, 0.93)** | **<.001** | **0.30 (0.01, 0.59)** | **.04** | **0.33 (0.03, 0.62)** | **<.001** |
| Photoperiod | – | – | – | – | **-0.04 (-0.04, -0.03)** | **<.001** |
| PTZ | – | – | -0.02 (-0.35, 0.32) | .93 | -0.02 (-0.35, 0.31) | .90 |
| Age | – | – | **-0.86 (-1.35, -0.36)** | **<.001** | **-0.85 (-1.34, -0.36)** | **.001** |
| Sex | – | – | **19.89 (17.28, 22.49)** | **<.001** | **19.99 (17.38, 22.59)** | **<.001** |
| *Ethnicity* |  |  |  |  |  |  |
| Arab/Middle Eastern | – | – | **-13.52 (-26.47, 0.58)** | **.04** | **-13.59 (-26.55, -0.63)** | **.04** |
| Asian | – | – | **-20.90 (-24.11, -17.68)** | **<.001** | **-20.97 (-24.19, -17.75)** | **<.001** |
| Black/African American | – | – | **-22.15 (-28.12, -16.18)** | **<.001** | **-22.23 (-28.20, -16.25)** | **<.001** |
| Latinx/Hispanic | – | – | **-7.18 (-14.57, 0.21)** | **.06** | -7.33 (-14.73, 0.07) | .05 |
| Multi-racial | – | – | -4.31 (-8.92, 0.30) | .07 | **-4.31 (-8.93, 0.31)** | .07 |
| Native American | – | – | 16.99 (-43.17, 77.15) | .58 | 17.51 (-42.72, 77.73) | .57 |
| Other | – | – | 1.70 (-16.45, 19.84) | .86 | -1.34 (-16.83, 19.50) | .89 |
| Surgical specialty | – | – | **-7.39 (-10.97, -3.82)** | **<.001** | **-8.30 (-10.94, -3.77)** | **<.001** |
| Days on internship | – | – | **0.02 (0.02, 0.02)** | **<.001** | **0.02 (0.02, 0.02)** | **<.001** |
| Sleep Midpoint (minutes after midnight) | – | – | **0.23 (0.23, 0.23)** | **<.001** | **0.23 (0.23, 0.24)** | **<.001** |
| Weekend | – | – | **17.21 (16.76, 17.67)** | **<.001** | **16.89 (16.62, 17.54)** | **<.001** |

Note. a. Sensitivity analyses including only individuals with ≥7 days of data in each 3-month interval: May, June, and July; August, September, and October; November, December, and January; February, March, and April. b. 95% confidence intervals were computed using the Wald method. c. PTZ = East–west position within time zone, calculated as the difference between institutional longitude and the central meridian of the time zone (e.g., –75° for Eastern, –90° for Central). d. Surgical specialty = binary assignment of surgical specialties were assigned based on the American College of Surgeons classification and included Neurological Surgery, Obstetrics and Gynecology, Ophthalmology, Orthopedic Surgery, Otolaryngology, Plastic Surgery, Surgery-General, Thoracic Surgery, Urology, and Vascular Surgery. Nonsurgical specialties included Anesthesiology, Child Neurology, Emergency Medicine, Family medicine, Internal Medicine, Internal Medicine/Emergency Medicine, Internal Medicine/Pediatrics, Internal Medicine/Psychiatry, Interventional Radiology, Neurology, Pathology-Anatomical and Clinical, Pediatrics, Pediatrics/Medical Genetics, Pediatrics/Psychiatry/Child and Adolescent Psychiatry, Physical Medicine and Rehabilitation, Psychiatry, Psychiatry/Family Medicine, Radiology-Diagnostic, Transitional Year. e. Sleep midpoint is the midpoint of sleep episode, expressed as minutes after midnight on the day the sleep episode ends.
