# Supplementary material for: Associations of latitude and photoperiod with sleep duration in a yearlong study of US physicians
Source: Sleep Med. Author manuscript; Available in PMC 2026 Jul 1. (PMC13322165; doi:10.1016/j.sleep.2025.106840)
Supplement: 4 [file NIHMS2184662-supplement-4.docx]

Table 2. LMMs without Sleep Midpoint as a Covariate

|  | Adjusted for covariates | | Adjusted for photoperiod and covariates | |
| --- | --- | --- | --- | --- |
| Variables | b (95% CI) | p | b (95% CI) | p |
| Latitude | **0.26 (0.05, 0.48)** | **.02** | **0.31 (0.09, 0.52)** | **.01** |
| Photoperiod | – | – | **-0.03 (-0.03, -0.02)** | **<.001** |
| PTZ | 0.03 (-0.22, 0.28) | .81 | 0.03 (-0.22, 0.28) | .81 |
| Age | **-1.01 (-1.34, -0.67)** | **<.001** | **-1.02 (-1.35, -0.69)** | **<.001** |
| Sex | **17.97 (16.23, 19.72)** | **<.001** | **17.93 (16.19, 19.67)** | **<.001** |
| *Ethnicity* |  |  |  |  |
| Arab/Middle Eastern | **-10.43 (-17.79, -3.07)** | **<.001** | **-10.41 (-17.77, -3.06)** | **.01** |
| Asian | **-17.97 (-19.79, -15.45)** | **<.001** | **-17.59 (-19.76, -15.42)** | **<.001** |
| Black/African American | **-21.93 (-25.89, -17.98)** | **<.001** | **-21.92 (-29.19, -20.77)** | **<.001** |
| Latinx/Hispanic | **-8.82 (-13.34, -4.31)** | **<.001** | **-8.75 (-13.26, -4.24)** | **<.001** |
| Multi-racial | **-3.70 (-6.74, -0.66)** | **.02** | **-3.65 (-6.69, -0.61)** | **.02** |
| Native American | -5.78 (-33.85, 22.28) | .69 | -4.50 (-32.52, 23.53) | .75 |
| Other | 3.15 (-11.54, 17.84) | .67 | 2.91 (-11.76, 17.57) | .70 |
| Surgical specialty | **-15.05 (-10.85, -5.87)** | **<.001** | **-14.88 (-17.29, -12.46)** | **<.001** |
| Days on internship | **0.02 (0.01, 0.01)** | **<.001** | **0.01 (0.01, 0.01)** | **<.001** |
| Weekend | **29.09 (16.53, 17.34)** | **<.001** | **29.09 (28.69, 29.50)** | **<.001** |

Note. a. 95% confidence intervals were computed using the Wald method. b. PTZ = East–west position within time zone, calculated as the difference between institutional longitude and the central meridian of the time zone (e.g., –75° for Eastern, –90° for Central). c. Surgical specialty = binary assignment of surgical specialties were assigned based on the American College of Surgeons classification and included Neurological Surgery, Obstetrics and Gynecology, Ophthalmology, Orthopedic Surgery, Otolaryngology, Plastic Surgery, Surgery-General, Thoracic Surgery, Urology, and Vascular Surgery. Nonsurgical specialties included Anesthesiology, Child Neurology, Emergency Medicine, Family medicine, Internal Medicine, Internal Medicine/Emergency Medicine, Internal Medicine/Pediatrics, Internal Medicine/Psychiatry, Interventional Radiology, Neurology, Pathology-Anatomical and Clinical, Pediatrics, Pediatrics/Medical Genetics, Pediatrics/Psychiatry/Child and Adolescent Psychiatry, Physical Medicine and Rehabilitation, Psychiatry, Psychiatry/Family Medicine, Radiology-Diagnostic, Transitional Year.
