# Supplementary material for: Associations of latitude and photoperiod with sleep duration in a yearlong study of US physicians
Source: Sleep Med. Author manuscript; Available in PMC 2026 Jul 1. (PMC13322165; doi:10.1016/j.sleep.2025.106840)
Supplement: 5 [file NIHMS2184662-supplement-5.docx]

Table 3. LMMs without Sleep Midpoint as a Covariate Among Interns with Broad Annual Coverage

|  | Adjusted for covariates | | Adjusted for photoperiod and covariates | |
| --- | --- | --- | --- | --- |
| Variables | b (95% CI) | p | b (95% CI) | p |
| Latitude | **0.35 (0.05, 0.48)** | **.01** | **0.37 (0.09, 0.65)** | **.01** |
| Photoperiod | – | – | **-0.03 (-0.03, -0.03)** | **<.001** |
| PTZ | 0.01 (0.07, 0.63) | .81 | 0.01 (-0.31, 0.32) | .97 |
| Age | **-0.99 (-1.44, -0.54)** | **<.001** | **-1.02 (-1.44, -0.54)** | **<.001** |
| Sex | **18.87 (16.48, 21.26)** | **<.001** | **17.93 (16.55, 21.33)** | **<.001** |
| *Ethnicity* |  |  |  |  |
| Arab/Middle Eastern | -6.41 (-18.23, 5.42) | .29 | -10.41 (-18.23, 5.41) | .29 |
| Asian | **-16.81 (-19.75, -13.86)** | **<.001** | **-17.59 (-19.78, -13.89)** | **<.001** |
| Black/African American | **-18.46 (-23.92, -13.01)** | **<.001** | **-21.92 (-23.95, -13.04)** | **<.001** |
| Latinx/Hispanic | -5.68 (-12.44, 1.07) | .10 | -8.75 (-12.54, 0.96) | .09 |
| Multi-racial | -3.29 (-7.50, 0.92) | .13 | -3.65 (-7.49, 0.93) | .13 |
| Native American | 17.96 (-36.86, 72.78) | .52 | -4.50 (-36.42, 73.18) | .51 |
| Other | 6.09 (-10.47, 22.65) | .47 | 2.91 (-10.71, 22.40) | .49 |
| Surgical specialty | **-14.13 (-17.49, -10.77)** | **<.001** | **-14.88 (-17.52, -10.79)** | **<.001** |
| Days on internship | **0.02 (0.02, 0.02)** | **<.001** | **0.01 (0.01, 0.02)** | **<.001** |
| Weekend | **29.11 (28.65, 29.57)** | **<.001** | **29.09 (28.65, 29.56)** | **<.001** |

Note. a. Sensitivity analyses including only individuals with ≥7 days of data in each 3-month interval: May, June, and July; August, September, and October; November, December, and January; February, March, and April. b. 95% confidence intervals were computed using the Wald method. c. PTZ = East–west position within time zone, calculated as the difference between institutional longitude and the central meridian of the time zone (e.g., –75° for Eastern, –90° for Central). d. Surgical specialty = binary assignment of surgical specialties were assigned based on the American College of Surgeons classification and included Neurological Surgery, Obstetrics and Gynecology, Ophthalmology, Orthopedic Surgery, Otolaryngology, Plastic Surgery, Surgery-General, Thoracic Surgery, Urology, and Vascular Surgery. Nonsurgical specialties included Anesthesiology, Child Neurology, Emergency Medicine, Family medicine, Internal Medicine, Internal Medicine/Emergency Medicine, Internal Medicine/Pediatrics, Internal Medicine/Psychiatry, Interventional Radiology, Neurology, Pathology-Anatomical and Clinical, Pediatrics, Pediatrics/Medical Genetics, Pediatrics/Psychiatry/Child and Adolescent Psychiatry, Physical Medicine and Rehabilitation, Psychiatry, Psychiatry/Family Medicine, Radiology-Diagnostic, Transitional Year.
